# A hypokalemic muscular weakness after licorice ingestion: a case report

**DOI:** 10.4076/1757-1626-2-8053

**Published:** 2009-09-17

**Authors:** Akkas Camkurt Meltem, Coskun Figen, Metin Aksu Nalan, Kunt Mahir, Bozkurt Sebnem, Isildak Mehlika, Kilic Ahmet Kasim, Bayraktar Miyase

**Affiliations:** 1 Department of Emergency, Hacettepe University, SÄ±hhÄ±ye Ankara, Turkey; 2 Department of Internal Medicine, Hacettepe University, SÄ±hhÄ±ye Ankara, Turkey; 3 Department of Neurology, Hacettepe University, SÄ±hhÄ±ye Ankara, Turkey

## Abstract

A 21-year-old male presented to the emergency department with the complaint of muscle weakness. The patient had used a powderized over-the-counter product named 'Tekumut' for 2 weeks to quit smoking. The granulated product was studied and determined to contain 'licorice' containing glycyrrhizic acid.

Licorice (a plant which contain glycyrrhizic acid) -induced hypokalemia usually has a mild progression. However, it may cause a critical failure in physical action by means of weakness followed by paralysis and may cause rhabdomyolysis or ventricular fibrillation, leading to death, when left untreated.

This report has presented the first case with hypocalemia due to licorice consumption in granulated form from Turkey. In addition, the report has aimed to emphasize the importance of obtaining the detailed history of a patient in diagnosis.

## Introduction

Weakness refers to a decrease in muscle strength. It is encountered in a number of medical and psychiatric disorders but it is highly a non-specific symptom. Therefore, evaluation and differential diagnosis of weakness in emergency room is often difficult and time consuming. Hypokalemia is one of the electrolyte problems that can lead to weakness. When hypokalemia is determined, underlying causes should be investigated.

Ingestion of Glycyrrhizic acid, which is the active component in licorice causes a metabolic syndrome mimicing primary hyperaldosteronism. Chronic intoxication with glycyrrhizic acid may cause hypertension, metabolic alkalosis and hypokalemia with low plasma renin activity. Licorice-induced hypokalemia usually has a mild progression. However, it may cause a critical failure in physical action by means of weakness followed by paralysis and may cause rhabdomyolysis or ventricular fibrillation, leading to death, when left untreated, [1].

Generally, the onset and severity of the symptoms depend on the dose and duration of licorice intake, as well as individual susceptibility [2].

## Case presentation

A 21-year-old patient Caucasian Turkish male presented to the emergency department with the complaint of weakness. He had played football one day before and noticed that he had difficulty in moving his extremities when he woke up at night. He had similar complaints a week ago while taking a shower and his complaint sustained for a few days. In his history of illness, he had double vision complaints and was evaluated for multiple sclerosis, but no diagnosis could be established four years ago.

On physical examination, his blood pressure was 140/80 mmHg, heart rate 86 beat/min, respiratory rate 18/min, and body temperature 36.7°C. The patient was alert, cooperative, and oriented. There was generalized paralysis in over all four limbs (power grade 3/5). There was no sensory deficit. Other systemic and neurologic examinations were normal.

The results of the laboratory evaluation were as follows: potassium (K^+^) 2.8 mEq/L and sodium (Na^+^) 141 mEq/L, urea 14 mg/dl, random blood glucose 110 mg/dl and creatinine 0.8 mg/dl. Blood gases showed pH 7.4, bicarbonate 22 mmol/L, pCO_2_ 31.3 mmHg, pO_2_ 97 mmHg and oxygen saturation 99%. Biochemical analyses of urine and thyroid function tests were normal. There was no specific finding at electrocardiogram.

The prediagnosis was thought to be a pseudo attack because of suspicion of multiple sclerosis related with double vision complaints in the past. Neurology department was consulted. However, cranial magnetic resonance imaging (MRI) did not confirm the prediagnosis. MRI and MRI angiography were normal.

During investigation period, initial therapy included 10 mmol of potassium chloride (KCL) per hour given by the intravenous route. Within two days, serum levels of potassium normalized and all clinical symptoms improved.

He was then asked for further history, it was recorded that about 2 months ago, the patient had used a powderized over-the-counter product named 'tekumut' for 2 weeks to quit smoking. He continued to use the same product at various intervals as he felt the desire to smoke. The patient was asked to bring a sample of the product and refrain from using it. The granulated product was studied and determined to contain 'licorice' containing glycyrrhizic acid.

The patient was discharged with recommendations of taking 1 tablet of oral potassium per day and applying to endocrinology and neurology clinics for follow up. The serum K^+^ level was normal after one week when he applied to the endocrinology outpatient clinic and he was asymptomatic. In the follow-up visit after 2.5 months, the serum potassium level of the patient was completely normalized and the patient was asymptomatic.

## Discussion

Licorice-induced hypokalemia is a rare condition, the most common symptom is generalized muscle weakness. Initial presentation with muscle paralysis is far more rare [3,4]. Licorice's active ingredient, glycyrrhizic acid inhibits 11 β-hydroxysteroid dehydrogenase, the renal enzyme which is responsible for conversion of cortisol to cortisone. As a result, renal mineralocorticoid receptors are activated by cortisol, which causes excess mineralocorticoid production [5]-[7]. Licorice-containing products are used in confectioneries, health products, chewing tobacco, chewing gums and in some alcoholic drinks. For treatment, licorice consumption should be restricted and potassium loss should be replaced. Potassium sparing diuretic, spironolactone, and dexamethasone administration should be considered. Dexamethasone reduces the cortisol-mediated stimulation of the mineralocorticoid receptor by suppressing endogenous cortisol production [5,8].

The mineralocorticoid stimulation by licorice is reversible, usually recovering within days, but may be sustained for several weeks according to amount taken and individual susceptibility [9].

In our patient, when obtaining the initial history, no history of licorice was recorded. The patient did not have vomiting, diarrhea, sweating and denied using drugs, including diuretics, alcohol, herbal medication, and laxatives. He was mildly hypertensive and had generalized muscle weakness. In laboratory evaluations, hypokalemia was determined. There was no other pathology. When the patient was asked about any drug and substance use for the second time, he said that he had been using a granulated over-the-counter herbal product which he bought in order to quit smoking. This is the first case of hypokalemia related to licorice consumption from Turkey. Thus, we could not expect or guess the content of the product. Still, he was asked to bring a sample of the product and quit using it. We determined that the active compound causing hypokalemia was Glycyrrhizic acid but were not able to determine its amount by analysis due to lack of technical support. In our country, the products which fall within the scope of vegetable based or herbal supplementary food are licensed by Ministry of Agriculture, but the amounts of active ingredients in those products are not under control. This product as well has been licensed by the Ministry of Agriculture; its content is defined, but the amount of active ingredients are not listed nor are standardized. The product invoved, as written on its directions and instructions, is said to be 100% herbal and it can be bought and used by anyone, upto 20 times a day, and no precautions or limitations are noted as well as the name of plants or their amounts. So we were not able to identify or tell the amount of licorice or Glycyrrhizic acid in the product.

It is already known that licorice consumption may cause undesirable mineralocorticoid-like side effects including hypertension, and hypokalemia. Similar symptoms and findings are also seen in Cushing's syndrome including ectopic ACTH syndrome and genetic diseases such as congenital adrenal hyperplasia, mineralocorticoid receptor abnormalities, Liddle syndrome, and the syndrome of apparent mineralocorticoid excess [10,11], although the possibility of these conditions could also be excluded because the patient was too old for congenital and genetic causes and had not experienced a similar disorder previously, in addition, his condition improved rapidly after the withdrawal of licorice containing 'tekumut' and replacement of K^+^.

## Conclusion

In this case, we realized that substances containing licorice, one of which is called 'tekumut', were being used in Turkey to quit smoking. In patients applying with hypokalemic paralysis, particularly when there is a clinical picture mimicking primary hyperaldosteronism, the use of such substances should be kept in mind.

## Patient perspective

I felt very weak and desperate. I couldn't walk and get up from bed. I want to quit smoking but I could die. So I will not take any medication or plant without consulting the doctor.

## Consent

Written informed consent was obtained from the patient for publication of this case report and accompanying images. A copy of the written consent is available for review by the Editor-in-Chief of this journal. You can find the copy of consent form next page.

## Competing interests

The authors declare that they have no competing interests.

## Authors' contributions

ACM was responsible overall preparation and revision of manuscript for intellectual concept, BS, CF helped in acquisition of data, MAN helped in preparation of the first draft and they were responsible for management of the patient. KM was responsible for conception of the idea. KAK differentiated other neurolojic diseases. IM and BM distinguish from other endocrinologic diseases. All authors read and approved the final manuscript.
